# Rapid diagnostics for *Gnomoniopsis smithogilvyi* (syn. *Gnomoniopsis castaneae*) in chestnut nuts: new challenges by using LAMP and real-time PCR methods

**DOI:** 10.1186/s13568-021-01266-w

**Published:** 2021-07-12

**Authors:** Anna Maria Vettraino, Nicola Luchi, Domenico Rizzo, Alessia Lucia Pepori, Francesco Pecori, Alberto Santini

**Affiliations:** 1 Department for Innovation in Biological, Agro-Food and Forest Systems (DIBAF), University of Tuscia, Via S. Camillo de Lellis Snc, 01100 Viterbo, Italy; 2 Institute for Sustainable Plant Protection, National Research Council (IPSP-CNR), Via Madonna del Piano 10, Sesto Fiorentino, 50019 Firenze, Italy; 3 Laboratory of Phytopathological Diagnostics and Molecular Biology, Plant Protection Service of Tuscany, Via Ciliegiole 99, 51100 Pistoia, Italy

**Keywords:** Chestnut rot, Portable diagnostics, Real-time quantitative PCR, LAMP, Isothermal amplification, Early detection

## Abstract

Nuts of the sweet chestnut (*Castanea sativa*) are a widely appreciated traditional food in Europe. In recent years producers and consumers reported a drop of nut quality due to the presence of rot diseases caused by *Gnomoniopsis smithogilvyi.* Early detection of this pathogen is fundamental to the economic viability of the chestnut industry. In the present study, we developed three molecular methods based on real-time portable LAMP, visual LAMP and qPCR assays for *G. smithogilvyi*. The molecular assays were specific for *G. smithogilvyi* and did not amplify the other 11 *Gnomoniopsis* species and 11 other fungal species commonly associated with chestnuts. The detection limit of both the qPCR and real-time portable LAMP (P-LAMP) assays was 0.128 pg/µL, while the visual LAMP (V-LAMP) assay enabled the detection up to 0.64 pg/µL. By using these newly developed molecular tools, the pathogen was detected in symptomatic and asymptomatic nuts, but not in leaves. The reliability of these molecular methods, including the P-LAMP assay, was particularly useful in detecting *G. smithogilvyi* of harvested nuts in field, even in the absence of rot symptoms.
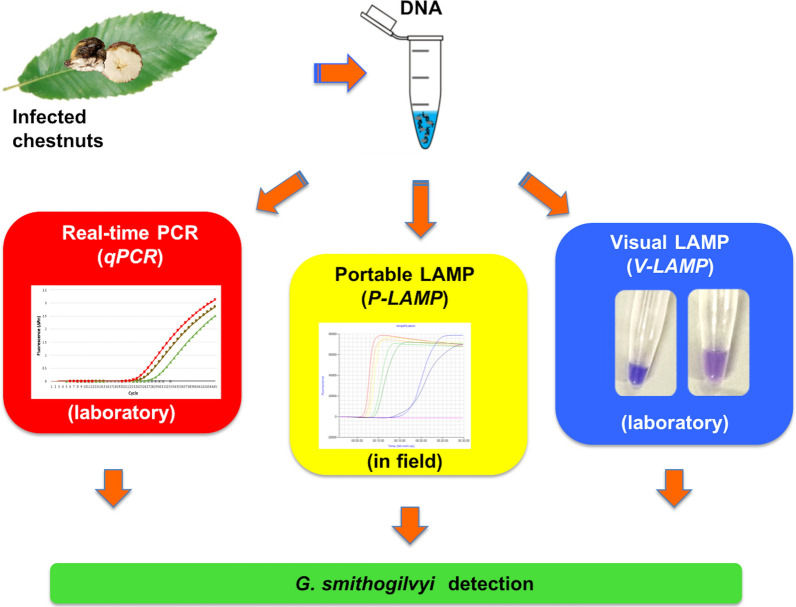

## Key points

- Three molecular methods have been developed to detect *G. smithogilvyi* from plant tissue
- These methods were specific and sensitive, even in asymptomatic samples
- These tools allow a rapid inspection of harvested chestnuts, in field and in lab

## Introduction

Chestnuts are a tree crop of high historical importance as a food source in several countries of the world. The first evidence of chestnut domestication dates back to prehistoric times in several geographic areas (Russell 1987; Keng 1974). In the third millennium BC, European chestnut (*Castanea sativa* Mill) that was native to the Caucasian-Armenian region, was slowly introduced by the Greeks and later the Romans to the west (Conedera et al. 2004). At that time chestnut was already appreciated as a multipurpose tree (Conedera et al. 2004; Meyer 1980). Chestnut fruits become one of the most important food resources of rural European mountainous areas. Unfortunately, during the twentieth century, the history of chestnut production underwent a period of crisis due to the impact of several plant diseases, which aggravated food shortages for rural populations and increased migration to urban areas. Only recently, customers reappraised nuts due to an increase in consumer awareness and interest about traditional foods beneficial to human health. Several studies highlighted the nutritional qualities and potentially valuable health effects of chestnuts. Chestnut is a low-fat fruit, rich in vitamins, minerals, sugars and several other compounds, including the ω-3 and ω-6 polyunsaturated fatty acids (PUFA) tocopherol and linoleic acid, that reduce a person's risk of developing diseases (Barreira et al. 2009; De Vasconcelos et al. 2010; Li et al. 2007; Rodrigues et al. 2020; Simopoulos 1991).

In the last century, global chestnut production has constantly been rising, reaching a volume of 2.4 metric tons (+ 18% in the last 5 years) (FAOSTAT 2021). Nevertheless, the fruit yield and quality can be seriously compromised by the attack of several fungi including *Acrospeira mirabilis*, *Alternaria* spp., *Aspergillus* spp., *Botrytis cinerea*, *Coniophora puteana*, *Cryptodiaporthe castanea*, *Cytodiplospora castanea*, *Discula campestris*, *Dothiorella* spp., *Fusarium* spp., *Neofusicoccum ribis*, *Penicillium* spp., *Pestalotia* spp., *Rhizopus* spp., *Sclerotinia sclerotiorum*, *Trichoderma* spp., *Trichothecium roseum*, just to name a few (Lione et al. 2019).

Some taxas as *Ciboria batschiana*, *Phoma* spp. and *Phomopsis* have been considered as particularly relevant in association with the spoilage or mummification of chestnut nuts (Maresi et al. 2013; Vettraino et al. 2005; Visentin et al. 2012; Washington et al. 1997).

In Italy, in the last decade a steep rise in the incidence of chestnut “pink rot” caused by *Colletotrichum acutatum* and black rot caused by *Gnomoniopsis smithogilvyi* has been reported. Currently, a low level of infection by *C. acutatum* has been recorded (Gaffuri et al. 2017). Conversely, *G. smithogilvyi* is considered the worldwide most important threat of chestnut fruits, present in Oceania, North America and Europe (EPPO Global database). In China, chestnut rot is caused by other species of *Gnomoniopsis*, *G. chinensis* and *G. daii* (Jiang et al. 2020; Jiang and Tian 2019).

*Gnomoniopsis smithogilvyi* and *G. castaneae,* although described independently from Europe and Australia, refer to a single species (Shuttleworth et al. 2015). However, the current name for this fungus is *Gnomoniopsois smithogilvyi*, while *Gnomoniopsis castaneae* is a synonym (Shuttleworth et al. 2018). Chestnut rot incidence caused by *G. smithogilvyi* has been reported to vary spatially and temporally, with peaks that can be as high as 91% observed in Switzerland (Dennert et al. 2015). *Gnomoniopsis smithogilvyi* has been found as an endophyte and is a common occurrence on several hosts such as *Castanea* spp., *Buxus sempervirens*, *Corylus avellana*, *Fraxinus ornus* and *Quercus ilex*, *Quercus cerris*, *Pinus pinaster* (Lione et al. 2019). On chestnut it has been mainly observed in association with bark cankers, twigs, leaves, nuts and galls induced by *D. kuriphilus* (Lione et al. 2019; Seddaiu et al. 2017; Vannini et al. 2017). The kernels of the affected chestnuts take on a burnished appearance with subsequent rotting of the nuts and an alteration of the organoleptic qualities resulting in significant economic losses.

Chestnuts are available mainly as fresh fruit, during the production season. The fruit has high-water content (around 50%), which compromises a long shelf life. To date, postharvest treatments, developed to minimize spoilage, can maintain the quality of chestnuts and extend their shelf life, but cannot completely stop the growth of pathogens (Vettraino et al. 2019, 2020; Lee et al. 2016; Kim et al. 2012). It is worth noticing that the efficacy of treatments is dependent on the level of the quality of nuts when they are harvested. It is a common practice among farmers to select chestnut consignments suitable for retailing or storage treatments on the basis of the percentage of rot incidence visually assessed. The pathogen detection based on visual inspection can lead to underestimation of the infection because *G. smithogilvyi* can live as endophyte within asymptomatic tissues (Maresi et al. 2013; Lione et al. 2019; Vannini et al. 2017).

Usually, the isolation and identification of a pathogen by culturing methods is time consuming and requires particular skills from the operator, especially if tissues can be colonized by different species belonging to the same genera. In addition, the isolation from these tissues may be difficult because the pathogen presence can be masked by other endophytic micro-organisms that overgrow them on agar media (Catal et al. 2001).

Pathogen detection, identification and quantification are critical steps for plant disease control and should be user-friendly to ensure food safety and sustainable crop production. In recent years several molecular methods based on real-time quantitative PCR (qPCR) have been used for a rapid detection of plant pathogens even in symptomless tissue (Abdullah et al. 2018; Hariharan and Prasannath 2021; Ioos et al. 2019; Luchi et al. 2006, 2020). More recently the loop mediated isothermal amplification (LAMP) has been developed through the use of relatively low-cost and portable devices for a rapid pathogen detection in field (Enicks et al. 2020; Luchi et al. 2020; Zhang et al. 2019). To our knowledge, no rapid and portable molecular methods have been developed for the detection of *G. smithogilvyi* in chestnut nuts tissues.

The aim of this study was to develop rapid, sensitive and reliable molecular methods based on LAMP and qPCR assays for the early detection of *G. smithogilvyi* on chestnut nuts by using both portable and laboratory instruments.

## Materials and methods

### Fungal isolates and DNA extraction

Eight strains of *Gnomoniopsis smithogilvyi*, 11 strains of different *Gnomoniopsis* species and 11 of other fungal species that commonly are associated to rot of chestnut fruits (Table 1) were obtained from the culture collections of KNAW-CBS (Westerdijk Fungal Biodiversity Institute, Utrecht, The Netherlands), DIBAF (University of Tuscia, Italy) and IPSP-CNR (Sesto Fiorentino, Italy). Isolates of *G. smithogilvyi* from infected *C. sativa* nuts (strains G1, G2, G3, G6, G7, G8) and *G. smithogilvyi* from CBS collection (CBS 130.190; CBS 142.044) were considered as positive reference samples (Table 1).

**Table 1:** List of fungal isolates used in this study

Fungal species	Strain	Host	Origin	Culture collection^a^
*Gnomoniopsis smithogilvyi* (syn. *G. castaneae*)	CBS 142.044	*Castanea sativa*	Vaud, Switzerland	CBS-KNAW
*G. smithogilvyi*	CBS 130.190	*Castanea* sp.	New South Wales, Australia	CBS-KNAW
*G. smithogilvyi*	G1	*C. sativa*	Viterbo, Italy	DIBAF
*G. smithogilvyi*	G2	*C. sativa*	Viterbo, Italy	DIBAF
*G. smithogilvyi*	G3	*C. sativa*	Viterbo, Italy	DIBAF
*G. smithogilvyi*	G6	*C. sativa*	Viterbo, Italy	DIBAF
*G. smithogilvyi*	G7	*C. sativa*	Viterbo, Italy	DIBAF
*G. smithogilvyi*	G8	*C. sativa*	Viterbo, Italy	DIBAF
*G. alderulensis*	CBS 125.680	*Rubus parviflorus*	Oregon, USA	CBS-KNAW
*G. chamaemori*	CBS 804.79	*Rubus chamaemorus*	Kevo, Finland	CBS-KNAW
*G. clavulata*	CBS 121.255	*Quercus falcata*	Maryland, USA	CBS-KNAW
*G. comari*	CBS 806.79	*Comarum palustre*	Lami, Finland	CBS-KNAW
*G. fructicola*	CBS 208.34	*Fragaria* sp.	Chevreuse, France	CBS-KNAW
*G. idaeicola*	CBS 125.676	*Rubus procerus*	Washington, USA	CBS-KNAW
*G. macounii*	CBS 121.468	*Spiraea* sp.	New York, USA	CBS-KNAW
*G. occulta*	CBS 125.678	*Potentilla* sp.	Oregon, USA	CBS-KNAW
*G. paraclavulata*	CBS 123.202	*Quercus alba*	Maryland, USA	CBS-KNAW
*G. racemula*	CBS 121.469	*Epilobium angustifolium*	Minnesota, USA	CBS-KNAW
*G. tormentillae*	CBS 904.79	*Quercus robur*	Vorden, Netherlands	CBS-KNAW
Other fungi				
* Alternaria* sp.	Fag8A1	*Fagus sylvatica*	Firenze, Italy	IPSP-CNR
* Botrytis* sp.	Pcyn22	*Protea cynaroides*	Firenze, Italy	IPSP-CNR
* Cladosporium* sp.	Gr2r23	*Quercus suber*	Grosseto, Italy	IPSP-CNR
* Cytospora* sp.	Li7r70	*Quercus suber*	Livorno, Italy	IPSP-CNR
* Diaporthe* sp.	Si1r47	*Quercus suber*	Siena, Italy	IPSP-CNR
* Diplodia seriata* sp.	Vtin20C	*Viburnum tinus*	Firenze, Italy	IPSP-CNR
* Epicoccum* sp.	Csemp5D	*Cupressus sempervirens*	Firenze, Italy	IPSP-CNR
* Fusarium* sp.	Fag8F	*Fagus sylvatica*	Firenze, Italy	IPSP-CNR
* Penicillium* sp.	18L2	*Quercus suber*	Firenze, Italy	IPSP-CNR
* Pestalotiopsis* sp.	Li1f71	*Quercus suber*	Livorno, Italy	IPSP-CNR
* Trichoderma* sp.	Li7r57	*Quercus suber*	Livorno, Italy	IPSP-CNR

^a^CBS-KNAW = Westerdijk Fungal Biodiversity Institute, Utrecht, The Netherlands; DIBAF = Department for Innovation in Biological, Agro-food and Forest systems (DIBAF)—University of Tuscia, Viterbo, Italy; IPSP-CNR = Institute for Sustainable Plant Protection -National Research Council, Sesto Fiorentino, Italy

Fungal isolates were grown on 300PT cellophane discs (Celsa, Varese, Italy) on potato dextrose agar (PDA; Difco Laboratories, Detroit, MI) in 90-mm Petri dishes and incubated in the dark at 20 °C. After 7 days, the mycelium was scraped from the surface of the cellophane and stored in 1.5-mL microfuge tubes (Sarstedt, Verona, Italy) at − 20 °C. Genomic DNA (gDNA) was extracted using the DNeasy plant mini kit™ (Qiagen, Courtaboeuf, France) following the manufacturer’s guidelines, after grinding mycelium with a Lysis matrix A tube containing one 6-mm ceramic sphere and garnet matrix (MP Biomedicals, Santa Ana, CA, USA) and homogenized for 20 s at 6.5 U (m/sec) using a FASTprep 24 device (MP Biomedicals) (Ioos et al. 2019). The concentration of extracted DNA was measured using a Nanodrop ND-1000 spectrophotometer (NanoDrop Technologies, Wilmington, DE, USA).

### Plant material and *G. smithogilvyi* isolation

Fresh nuts and leaves of *Castanea sativa* were randomly collected in Monti Cimini, Viterbo, Italy (800 m a.s.l, 42° 21′ 29.52″ N; 12° 10′ 39.72″ E) at harvest time in September 2020. Upon arrival at the laboratory, samples were surface sterilized as described by Luchi et al. (2006) (75% ethanol (1 min), 3 min in 3% NaClO and 75% ethanol (30 s) rinsed three time with sterile distilled water). Kernels were cut on half and visually assessed for the presence of decay. A total of 40 nuts (20 asymptomatic and 20 showing rots) and 20 leaves were split into two portions: one used for conventional isolation on agarized nutrient media and the second for DNA extraction using DNeasy plant mini kit (Qiagen), as described above. The portion of plant samples used for the pathogen isolation was cut in small fragments (5 × 5 mm) and plated in 90 mm Petri dishes containing PDS (39 g/L potato dextrose agar-PDA, Oxoid, UK) amended with streptomycin sulfate (0.06 g/L) as described by Vettraino et al. (2019). Plates were incubated at 25 °C for 4 days, and fungal colonies that morphologically resembled *G. smithogilvyi* were sub-cultured in new agar media for DNA extraction as described above. Molecular identification of *G. smithogilvyi* was conducted according to Vannini et al. (2017) using b-tubulin, the elongation factor 1-a (*EF1-a*), RNA polymerase II (rpbII) and the internal transcribed spacer (ITS) genes.

Over the 40 nuts and 20 leaves plated, a total of 20 nuts (10 symptomatic and 10 asymptomatic) and 10 leaves were selected for further molecular analysis, considering as “true positives” symptomatic nut samples *G. smithogilvyi* positive at culturing (TP; 10 samples) and as “true negatives” asymptomatic nut samples *G. smithogilvyi* negative at culturing (TN; 10 samples).

### Primer and probe design

Sequence data of the elongation factor 1-alpha (*EF1-α*) gene for *G. smithogilvyi* and other related species were obtained from the National Center for Biotechnology Information (NCBI) database (www.ncbi.nlm.nih.gov). Sequences were aligned using the T-Coffee package (Notredame et al. 2000). Regions conserved among all tested *G. smithogilvyi* populations but differentiating between closely related fungal species were chosen for LAMP and qPCR primer and probe design.

The *G. smithogilvyi* TaqMan® MGB probes and primer (Table 2) were designed using the Primer Express® Software 3.0 (Applied Biosystems, Foster City, CA, USA) on the basis of the same previously described sequences (KR072534). The TaqMan® MGB probe was labeled with 6-carboxy-fluorescein (FAM) at the 5′ end and a non-fluorescent quencher (NFQ) with minor groove binder (MGB) ligands as quencher, at the 3′ end.

**Table 2:** *Gnomoniopsis smithogilvyi* LAMP and qPCR primers and probe designed on elongation factor 1-alpha (*EF1-α*) gene

Molecular assay	Primer/probe name	Sequence (5′–3′)	Length (bp)
LAMP	Gno_F3	TTGTCGCTCTTATCTGGGA	19
	Gno_B3	CCTTCAGCTTGTCCAGAA	18
	Gno_LoopF	GGCTGGTGAGGTTGATGG	18
	Gno_LoopB	CTGCTGAGCTCGGAAAGG	18
	Gno_FIP	TGGGGTGGAGTTCTAGGCATCTTTACCGTCACTCCTTGG	39
	Gno_BIP	GTTGTCATGATCTCGTCGCTGACCCAGGCGTACTTGAAAG	40
qPCR	Gno_Fw	GCGGGAGTGGCTCGTCTT	18
	Gno_Rev	GCACCACCGAAAACGAAAA	19
	Gno_Probe^a^	***FAM***-TTTCGCCTGCTGCACA-***MGBNFQ***	21

^a^TaqMan MGB dual labeled-probe: 5′ *FAM* = 6-carboxyfluorescein; 3′ *MGBNFQ* = Minor Groove Binder (MGB) and Non-Fluorescent Quencher (NFQ)

The six *G. smithogilvyi* LAMP primers (Table 2) included: two outer primer (forward primer, F3; backward primer, B3) two inner primers (forward inner primer, FIP; backward inner primer, BIP) and two loop primers (forward loop primer, FLP; backward loop primer, BLP), as required by LAMP reaction (Notomi et al., 2000). Primers were designed using LAMP Designer software (OptiGene Limited, Horsham, UK) on the basis of the consensus sequences of the *EF1-α* gene (accession n. KR072534; strain CBS 130190) derived from the NCBI database.

The identity of the LAMP and qPCR amplicon sequences with other fungal species was determined with the Standard nucleotide-nucleotide BLAST (blast n) of the NCBI (Altschul et al. 1990).

All designed LAMP and qPCR primers were synthesized by Eurofins Genomics (Ebersberg, Germany), while the TaqMan® MGB probe was provided by Life Technologies (Paisley, UK). Primer and probe specificity were also tested in LAMP and qPCR assays on DNA from axenic cultures listed in Table 1.

### Diagnostic methods for *G. smithogilvyi*

Three different methods were optimized in this work for *G. smithogilvyi*: i) real-time quantitative PCR (qPCR); ii) visual LAMP (V-LAMP), and iii) portable real-time LAMP (P-LAMP) assays using a field instrument. Reactions sets included all fungal species reported in Table 1 and no template controls (NTC, with ddH_2_0). Reactions were assayed in duplicate.

#### Real-time quantitative PCR (qPCR) assay

DNA extracts from mycelium and plant tissues, were assayed in MicroAmp Fast 96-well Reaction Plates (0.1 mL) closed with optical adhesive, and using the StepOnePlus™ Real-Time PCR System (Applied Biosystems, Life Science, Foster City, CA, USA).

Real-time qPCR was performed in a 25 μL final volume containing 12.5 μL TaqMan Universal Master Mix, 300 nM forward primer, 300 nM reverse primer, and 200 nM TaqMan MGB probe and 5μL genomic DNA.

The PCR protocol was 50 °C (2 min), 95 °C (10 min), 50 cycles of 95 °C (30 s), and 60 °C (1 min).

Results were analyzed using an SDS 1.9 sequence detection system (Applied Biosystems) after manual adjustment of the baseline and fluorescence threshold. Quantification of *G. smithogilvyi* DNA in unknown samples was made by interpolation from a standard curve generated with a reference DNA standard (sample CBS 130190) that was amplified in the same PCR run. The standard curve was generated from ten 1:5 serial dilutions (ranging from 10 ng to 5.12 fg/µL) of a known concentration of *G. smithogilvyi* DNA and analyzed in triplicate. Reproducibility of the qPCR assay was assessed by computing the coefficient of variation (CV) among the mean values in eight independent assays. PCR efficiency was calculated on the slope of the standard curve (Eff = 10^–1/slope −1^) (Bustin et al. 2009), from eight independent experiments. The amount of *G. smithogilvyi* DNA from chestnuts tissues was indicated as picograms of fungal DNA per microgram of total DNA extracted.

#### Portable real-time LAMP (P-LAMP) assay

Real-time LAMP reactions were performed and optimized on the portable real-time fluorometer Genie ® II (OptiGene Limited, Horsham, UK). Each isothermal amplification reaction mixture contained 15 μL Isothermal Master Mix (ISO-001) (OptiGene Limited, Horsham, UK), 7 μL LAMP primer mixture (at final concentrations 0.2 μM of each F3 and B3, 0.4 μM of each FLP and BLP and 0.8 μM of each FIP and BIP) and 3 μL of template DNA.

The LAMP amplification reaction was run at 64 °C for 30 min, followed by an annealing analysis from 98 to 80 °C with ramping at 0.05 °C per second that allow the generation of derivative melting curves (Abdulmawjood et al. 2014). Reactions were performed in duplicate. The positivity of a sample was assessed on the base of amplification time (t_amp_) and amplicon annealing temperature (T_a_). The t_amp_ is the time (expressed in min) where the fluorescence second derivative of the signal reaches its peak above the baseline value, while the T_a_ is the temperature (expressed in °C) at which double-stranded DNA product dissociates into single strands.

#### Visual LAMP (V-LAMP) assay

Visual LAMP reactions were conducted in 25 μl volumes by varying the following compounds: 10 × primer mixture (2.5–4.0 µl), concentrations of the different LAMP primers (0.2–0.4 µM of F3/B3, 0.4–0.8 µM of LoopF/LoopB, and 0.8–1.2 µM of FIP/BIP, amount of the 10X isothermal buffer from New England Biolabs (2–4 µL), dNTPs (0.4–1.0 mM), Betaine (0.2–0.6 M), MgSO4 (2–6 mM), hydroxynapthol blue (HNB; 100–300 µM) and 8U Bst DNA polymerase from New England Biolabs (0.32–0.64 U/µL). To each reaction 2 ul of DNA template was added. The mixture was incubated at 64 °C for 30–40 min, followed by heating at 80 °C for 2–4 min to terminate the reaction.

The reactions were optimised at 2.5 µL 10 × Isothermal Buffer, 0.6 mM of dNTPs, 2 mM of MgSO_4_, 0.2µL of HNB 20 mM, 0.2 M of betaine, 2.0 µL 10 × LAMP primer mixture (0.2µL of each of the F3 and B3, 0.4 µL of each of LoopF and LoopB and 0.8 µL of each of the FIP and BIP primers), 8U of Bst polymerase and 2ul of template DNA. Reactions were performed at 64 °C per 30 min, followed by an additional cycle of 80 °C for 3 min. Products were kept at 4 °C. LAMP positive reactions to *G. smithogilvyi* were evaluated by a visual detection of the products by watching the color changes occurring in the tubes from purple (negative reaction) to blue (positive reaction). LAMP products were also subjected to agarose gel electrophoresis analyzed by gel electrophoresis on 3.0% agarose gel.

### Limit of detection (LOD) of LAMP and qPCR assays

To evaluate the limit of detection of each molecular method (qPCR, P-LAMP and V-LAMP assays), ten 1:5 serial dilutions ranged between 10 ng to 5.12 fg/µL of reference *G. smithogilvyi* DNA (strain CBS 142044, Table 1) were analyzed. The amplification products of V-LAMP were also confirmed through an agarose gel electrophoresis. Each specimen was tested in duplicate.

### Validation of LAMP and qPCR assays in chestnut tissue

To assess the effectiveness of DNA extraction, all DNA plant samples were further tested using a previously developed LAMP assay with cytochrome oxidase (*COX*) gene primers, as an internal positive control (Tomlinson et al. 2007). The reliability of the methods (qPCR, P-LAMP and V-LAMP) to detect *G. smithogilvyi* in naturally infected plants was tested by using DNA isolated from 10 symptomatic nuts (TP), 10 asymptomatic nuts (TN) and 10 leaves obtained as described above. True positives and true negatives samples were analyzed by the molecular assays described above. In this study culturing data have been collected and considered as “gold standard” (Anonymous 2004, 2006; Vettraino et al. 2005) to better evaluate the presence of false positives analysis detected by the newly developed molecular methods.

The diagnostic sensitivity and the diagnostic specificity of the both molecular and culturing methods was tested, in accordance with the EPPO standards on diagnostics (EPPO 2017, 2019): percent diagnostic sensitivity = (A/(A + C) × 100); percent diagnostic specificity = (D/(B + D × 100); percent diagnostic accuracy = A + D/(A + C + D + B) × 100, where A = obtained positives/expected positives (true positives); B = obtained negatives/expected positives (false negatives); C = false positives and D = true negatives.

## Results

### Fungal isolation from chestnuts samples

Molecular identification of mycelium isolated from nuts confirmed the presence of *G. smithogilvyi* in processed samples. A total of 60 samples of chestnut tissues (40 nuts and 20 leaves) were plated on PDS to check the presence of *G. smithogilvyi*. The pathogen was isolated both from symptomatic (18 out of 20 necrotic nuts; 90% of the samples) and asymptomatic chestnuts (9 out of 20 apparently healthy nuts; 45% of the samples). No *G. smithogilvyi* strains were obtained from 20 collected chestnut leaves.

### Specificity and sensitivity of LAMP and qPCR assay

The BLAST search showed a significant identity of the LAMP (Percentage of Identity = 100%; E-value = 9e−74) and qPCR (Percentage of Identity = 100%; E-value = 1e−18) amplicon sequences only with *G. smithogilvyi* strains. No significative homology was found with any species other than *G. smithogilvyi*.

The specificity of molecular assay was confirmed by testing DNA extracted from mycelium, belonging to different fungal species, hosts and geographical regions (Table 1). The qPCR, P-LAMP and V-LAMP assays always yielded positive results with DNA samples from 8 *G. smithogilvyi* strains and always negative results with all other 11 *Gnomoniopsis* species and other 11 fungal species (Table 1).

**Fig. 1:**
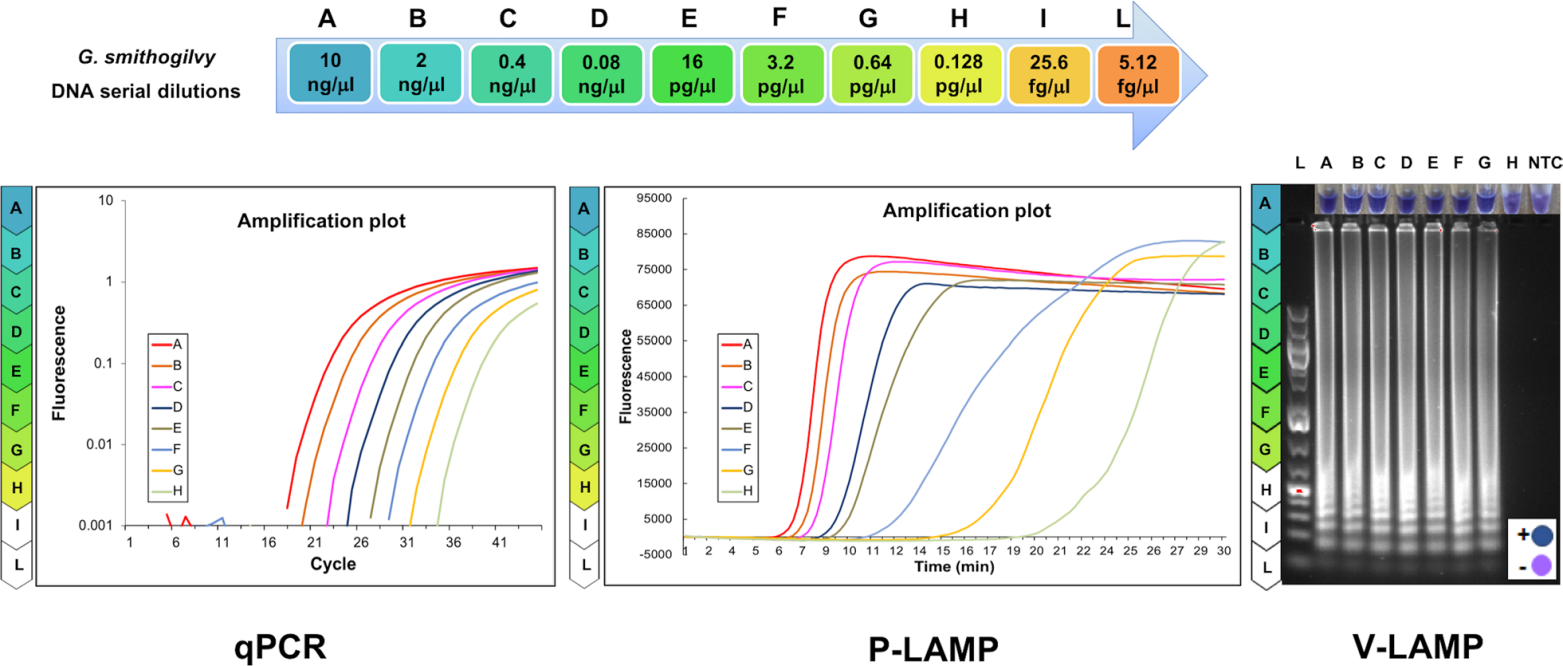
Comparison of sensitivity among qPCR (left), P-LAMP (middle), V-LAMP (right) assays using ten 1:5 serial dilutions ranged between 10 ng and 5.12 fg/µL of reference *Gnomoniopsis smithogilvyi* DNA (strain CBS 142044). The assessment for the V-LAMP assay was based on the HNB color change visualization of the LAMP products (Positive samples = blue color change in reaction, negative samples = violet color reaction) and based on gel electrophoresis: L-1000 bp ladder, Dilutions A-G showed positive reactions while no amplification was observed in dilution H and NTC (No Template Control)

The limit of detection (LOD) to recognize *G. smithogilvyi* for both qPCR and P-LAMP assays was 0.128 pg/µL (Fig. 1). The qPCR standard curve for *G. smithogilvyi* exhibited a slope of − 3.37, an R^2^ coefficient of 0.99 and a Y-intercept of 34.32. Efficiency of the qPCRs was 0.98 ± 0.04 (SD). Reproducibility of the standard curve points performed on eight curves was high (CV varied from 1.1 to 1.7%).

**Fig. 2:**
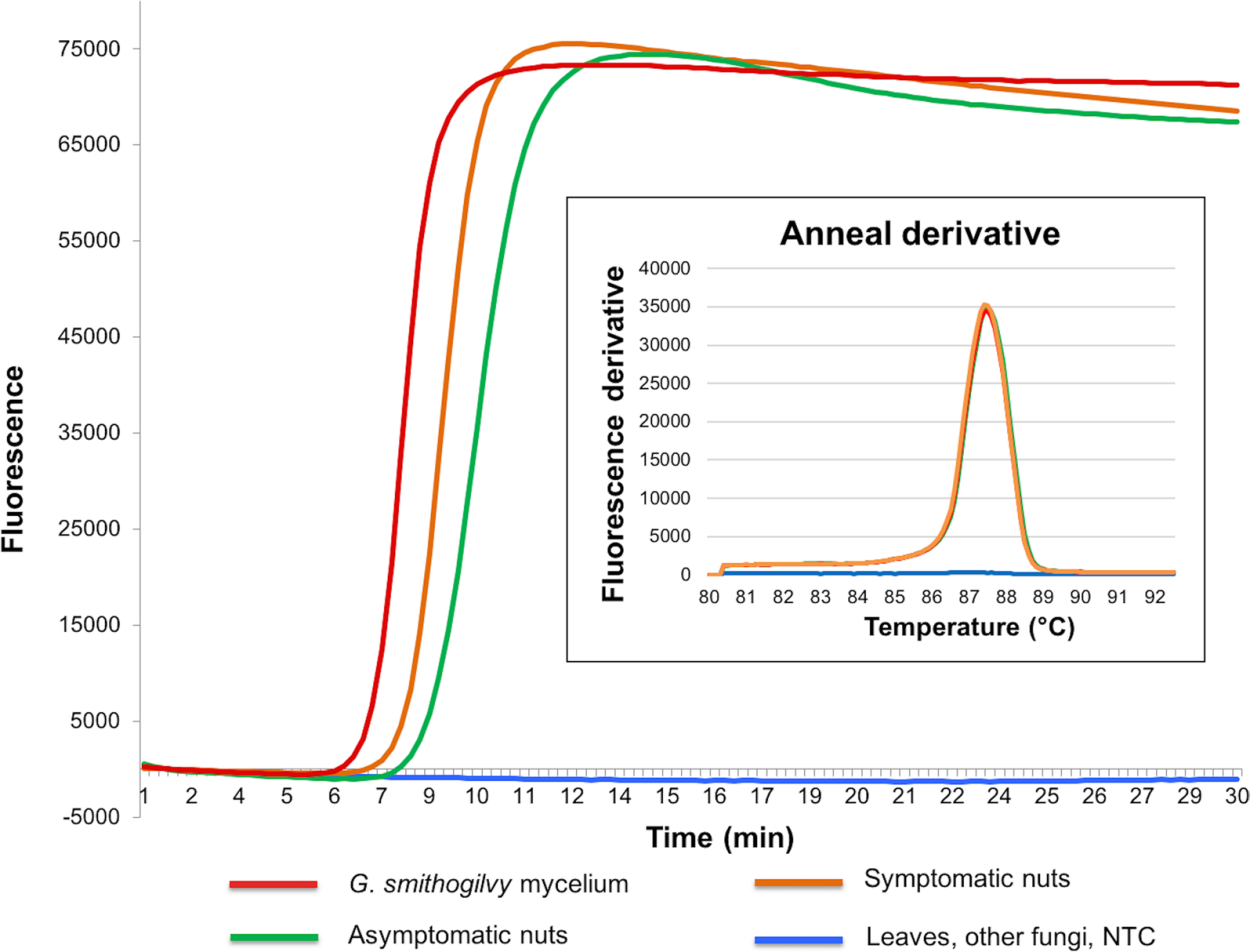
Portable LAMP (P-LAMP) by Genie II to detect *Gnomoniopsis smithogilvyi* DNA in symptomatic and asymptomatic nuts in comparison with fungal mycelium. The insert shows the specific *G. smithogilvyi* melting peak (T_a_ = 87.37 °C)

In P-LAMP a single melting peak with characteristic temperature (87.37 ± 0.05 °C) was observed for all *G. smithogilvyi* strains allowing identification of the pathogen (Fig. 2).

The LOD for V-LAMP was 0.64 pg/µL, showing that this last assay is less sensitive (Fig. 1). Positive reactions of the V-LAMP assay were represented by a colour shift from violet to blue of the reaction solution. The sensitivity of inspection by the naked eye was confirmed by detection using electrophoresis gel (Fig. 1). The nuclease-free water templates showed no colour change in any validation test.

### Fungal detection in chestnut samples

For each analyzed DNA plant sample (both asymptomatic (TN) and symptomatic (TP) chestnut tissues) LAMP analyses by *COX* gene amplification, showed a specific melting peak at the annealing temperature (T_a_ = 85 °C).

**Fig. 3:**
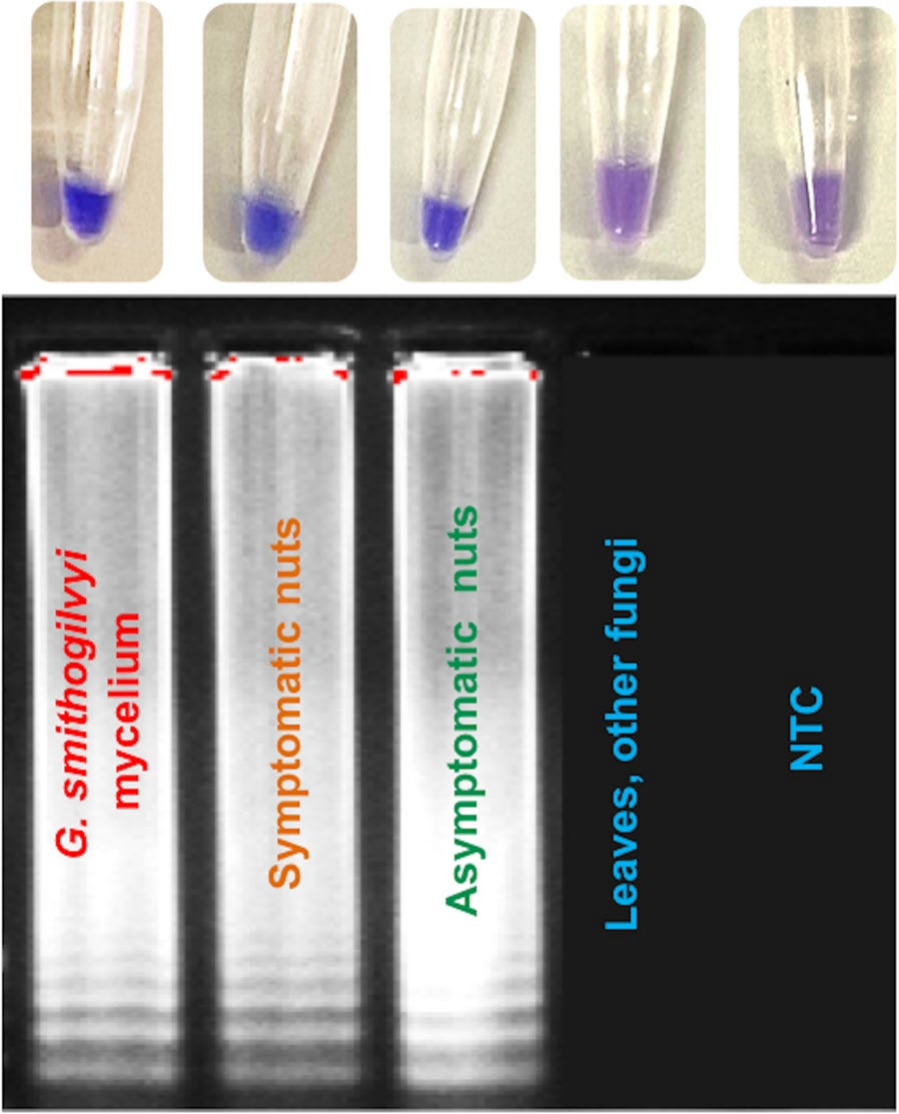
Visual detection of LAMP products (V-LAMP) by single-tube colorimetric reaction and electrophoretic gel. Representative visual inspection of *Gnomoniopsis smithogilvyi* from different plant tissues and mycelium. NTC = No Template Control. The assessment for the V-LAMP assay was based on the HNB color change visualization of the LAMP products (Positive samples = blue color change in reaction; negative samples = purple color reaction)

**Fig. 4:**
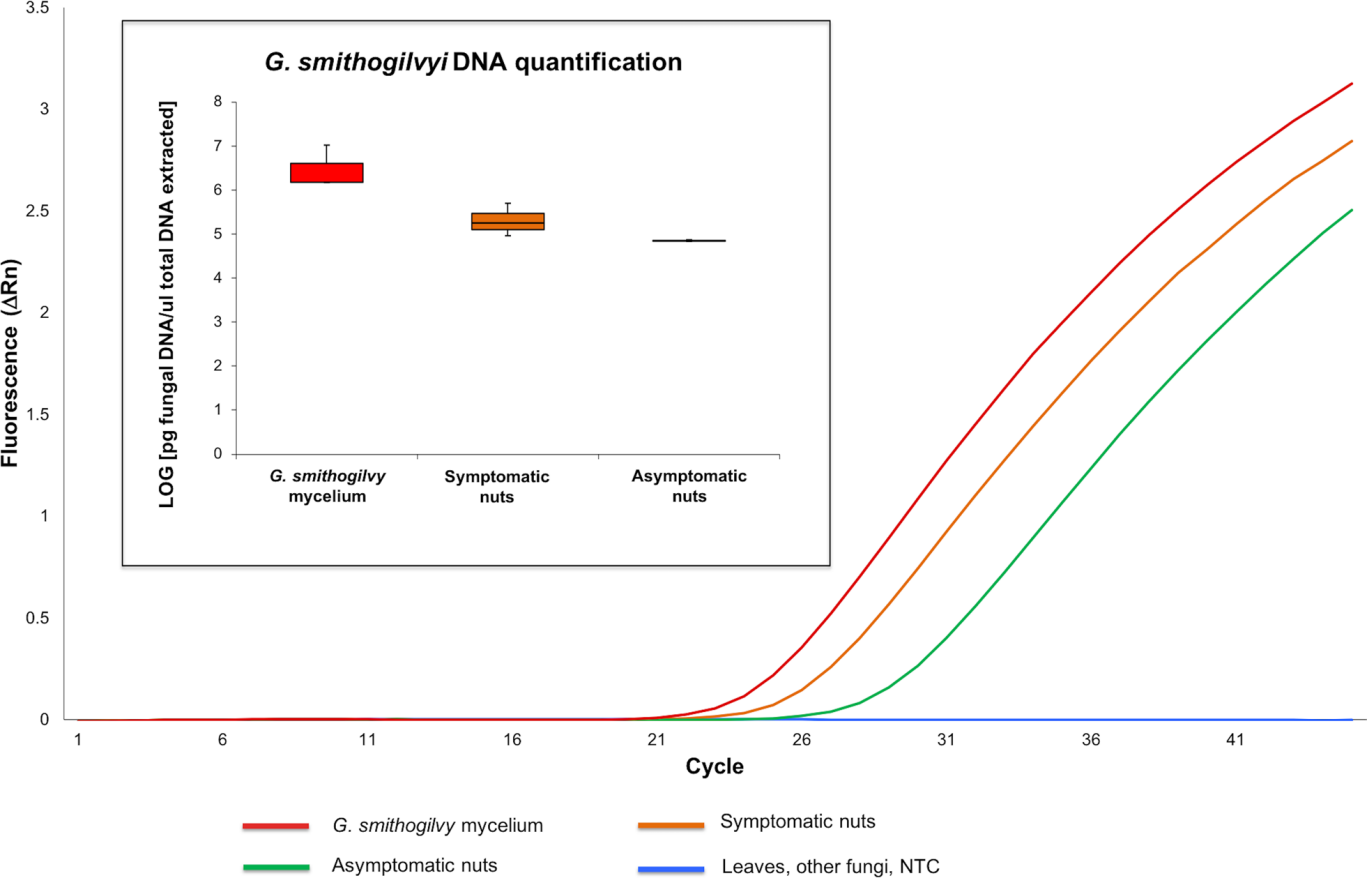
Amplification plots of *Gnomoniopsis smithogilvyi* DNA in chestnut and mycelium samples using qPCR (TaqMan MGB probe). The box-plots inset show the quantification of fungal pathogen DNA in plant tissues (symptomatic and asymptomatic) in comparison with *G. smithogilvyi* mycelium from axenic culture. NTC = No Template Control

The qPCR, P-LAMP and V-LAMP methods also detected and quantified *G. smithogilvyi* DNA from all symptomatic (10) and asymptomatic (10) nut samples, while in the leaves (10) *G. smithogilvyi* was not found (Figs. 2, 3, 4).

By using the P-LAMP assay, in symptomatic nuts the amplification signal was earlier (t_a_ = 8 min) than asymptomatic tissues (t_a_ = 10 min). Both symptomatic and asymptomatic nuts showed a specific melting peak to those found in DNA from *G. smithogilvyi* mycelium (T_a_ = 87.37 °C), confirming the specificity of the P-LAMP assay to detect the pathogen in infected nuts (Fig. 2). These results were also confirmed by V-LAMP assay (Fig. 3).

After quantification of *G. smithogilvyi* by qPCR, the amount of positive fungal DNA results was higher in symptomatic nuts (ranged between 1.7 × 10^5^ to 4.9 × 10^5^ pg DNA per µg of total DNA extracted), in comparison to symptomless nuts (ranged between 6 × 10^4^ to 7.5 × 10^4^ pg DNA per µg of total DNA). The quantification of DNA from mycelium showed the highest amount (1.5 × 10^6^ and 1.07 × 10^7^ pg DNA per µg of total DNA) (Fig. 4). The data trend was confirmed by using the P-LAMP assay (Fig. 2).

The qPCR, P-LAMP and V-LAMP showed identical values of diagnostic parameters (diagnostic sensitivity = 100%; diagnostic specificity = 100%, diagnostic accuracy = 100%). However when the analysis included the culturing data some values decrease (diagnostic sensitivity = 50%; diagnostic specificity = 100%, diagnostic accuracy = 67%), due to the presence of ‘false negatives’.

## Discussion

In the recent years, brown rot symptoms associated with *G. smithogilvyi* greatly impacted on the quality of nuts production with as consequence an important negative effect on the economics of chestnut growers. Therefore, the early detection of this pathogen is crucial for preventing and managing the disease with immediate practical outcome.

In this study we optimized three rapid and accurate diagnostic tools based on qPCR and loop-mediated isothermal amplification (V-LAMP and P-LAMP) to molecularly detect the presence of *G. smithogilvyi* in chestnuts symptomatic and asymptomatic tissue with lab and field tools (Dennert et al. 2015; Vettraino et al. 2019). Since pure culture is considered as the ‘gold standard’ in microorganisms detection and quantification, isolation of *G. smithogilvyi* has been used for the validation of molecular methods (Kralik and Ricchi 2017), because it ensure the presence of true positive. Nevertheless, the pathogen isolation is sometimes challenging, probably due to the low amount of the pathogen or the presence of other organisms growing faster on artificial substrates, it is generally time consuming, and needs great operator skills to isolate and identify the culture. Visentin et al (2012) reported a frequency of isolation from rotten chestnut nuts ranging from 35.0% (year 2009) to 74.6% (year 2011). In the present study*, G. smithogilvyi* was isolated both from rotten (90% of samples) and asymptomatic nuts (45% of the samples), confirming data from the literature (Lione et al. 2019).

The *G. smithogilvyi* molecular assays developed in this study (qPCR, P-LAMP and V-LAMP) were found to have high diagnostic statistics: accuracy, sensitivity and specificity measures reached values of 100%. The PCR efficacy, sensitivity for those methods, the absence of contaminations and the evaluation of DNA from *G. smithogilvyi* both in silico and in vivo analysis were in accordance with the guidelines defined by Broeders et al. (2014).

The correct design of primer and probe in a multiple copy rRNA gene resulted to be an important parameter in the development of very sensitive methods. The successful of amplification of both LAMP and qPCR primers and probe relies on specificities of these newly designed oligonucleotides. In addition, the MGB probe used in this work represents an improvement on the TaqMan probe with short oligonucleotides conjugated with a minor groove binder (MGB) at the 3′ end that increases the melting temperature (T_m_) of the probe and facilitates high reaction specificity and sensitivity (Kutyavin et al. 2000).

The pathogen was detected, without cross-react with DNA from other *Gnomoniopsis* species phylogenetically related or other fungal species widely present on nuts. Moreover, the efficacy in detecting low *G. smithogilvyi* DNA values, 0.128 pg/µL for qPCR and P-LAMP and 0.64 pg/µL for V-LAMP, confirms that the protocols developed can be applied for detection of the pathogen at low inoculum concentration. The presence of *G. smithogilvyi* was revealed even in asymptomatic tissues, negative at culturing, with a DNA concentration value (ranged between 6 × 10^4^ to 7.5 × 10^4^ pg DNA per µg of total DNA) tenfold lower than in symptomatic tissues (ranged between 1.7 × 10^5^ to 4.9 × 10^5^ pg DNA per µg of total DNA extracted). This finding is in agreement with previous studies on different pathogens where latent infections were detected in asymptomatic host tissues (Migliorini et al. 2015; Hughes et al. 2011; Vettraino et al. 2010).

Results gave evidence that the molecular approaches developed in this research can be useful for the detection of the pathogen before associated symptoms occurs, allowing farmers to applied specific protocols that can enhance the shelf life of the products.

Although all the assays developed in this study are reliable methods for the detection of *G. smithogilvyi*, practical reasons could justify their different application. Both the P-LAMP and qPCR assays are rapid, being able to detect *G. smithogilvyi* after short time (c.a. 30 min for P-LAMP and 90 min for qPCR). By using P-LAMP it possible to observe a positive fluorescent signal after only 8 min for symptomatic nuts and after 10 min for asymptomatic samples. The P-LAMP can be performed on a low consumption portable platform, suitable for use in the field, without the need for specially equipped laboratories. The use of a portable tool that delivers results quickly could be beneficial on farms, where a rapid assessment of the percentage of infected fruits could be used to make decisions related to storage and processing of chestnuts. Finally, the cost of each of the assays tested may be considered. The LAMP assay, with its requirement for specialized enzymes and reagents costs approximately 4 times more than real-time PCR. However, the LAMP instrument is cheaper than a real-time thermocyclers, especially if the V-LAMP is performed. The V-LAMP method was found to be the simplest method and positive amplicons can be observed with the naked eye with the addition of a dye in the reaction, eliminating the need of sophisticated instrumentations.

The new detection methods can be advantageous for a variety of applications. They are promising tools to make decisions relating to chestnuts storage and processing as well as to gain deeper insights into the biology of the pathogen increasing knowledge on the conditions trigging the fungus to shift from a latent to a pathogen lifestyle and opening opportunity to define control strategies.

## Data Availability

The datasets generated during and/or analysed during the current study are available from the corresponding author on reasonable request.
